# Impaired Aβ clearance: a potential link between atherosclerosis and Alzheimer’s disease

**DOI:** 10.3389/fnagi.2015.00115

**Published:** 2015-06-16

**Authors:** Ajay Gupta, Costantino Iadecola

**Affiliations:** ^1^Feil Family Brain and Mind Research Institute, Weill Cornell Medical CollegeNew York, NY, USA; ^2^Department of Radiology, Weill Cornell Medical CollegeNew York, NY, USA

**Keywords:** Alzheimer’s disease (AD), atherosclerosis, A beta peptide, dementia, vascular, A beta clearance

## Abstract

Alzheimer’s Disease (AD) and atherosclerosis remain two of the largest public health burdens in the world today. Although traditionally considered distinct pathological entities, mounting epidemiologic, clinical and experimental evidence suggests that cerebrovascular atherosclerosis and AD interact reciprocally to disrupt brain structure and function. Whereas the hypoperfusion and hypoxia caused by atherosclerosis of cerebral vessels may enhance the production of amyloid-β peptide (Aβ), a peptide central to AD pathology, Aβ, in turn, may promote formation of atherosclerotic lesions through vascular oxidative stress and endothelial dysfunction leading to additional vascular damage. Here, we briefly review evidence suggesting that impaired clearance of Aβ is an additional, simultaneously occurring mechanism by which AD and cerebrovascular disease may be causally linked. We examine the literature supporting mechanisms by which flow-limiting large-artery stenosis, arterial stiffening and microvascular dysfunction could contribute to AD pathophysiology by impairing Aβ clearance and elevating brain levels of Aβ. Finally, we highlight the need for further research to improve our understanding of the complex interactions of AD and atherosclerosis with Aβ clearance, which may ultimately serve to guide the development of novel diagnostic and therapeutic approaches for this devastating and highly prevalent condition.

## Introduction

Alzheimer’s Disease (AD) remains a major cause of morbidity and mortality worldwide. Recent estimates suggest that the public health burden attributable to AD will only continue to increase, with over 100 million AD cases projected worldwide by the year 2050 (Brookmeyer et al., 2007). Despite evidence that the prevalence of AD has declined in the last two decades (Larson et al., 2013), the anticipated expansion of the aging population is predicted to offset such decline. As such, there is a pressing need to develop new approaches to mitigate the enormous societal impact of AD-related dementia. To this end, an important strategy is pinpointing specific pathophysiologic mechanisms involved in AD development, including the identification of potentially modifiable risk factors commonly seen in aging populations (Norton et al., 2014).

One such potential risk factor strongly associated with the development and progression of AD is atherosclerosis of the cerebral arteries. Atherosclerosis has long been implicated in the pathogenesis of dementia, including theories existing since the early 1900s in which “hardening of the arteries” was hypothesized to play a central role in the development of dementia (Iadecola, 2013). With the identification in the 1980’s of amyloid-β peptide (Aβ) as the main constituent of amyloid deposits in the cerebral vasculature (amyloid angiopathy) and in the brain parenchyma (amyloid plaque) in AD (Glenner and Wong, 1984; Kang et al., 1987), the emphasis shifted towards AD neuropathology as the major cause of dementia (Bowler, 2007). Subsequent investigations of the association between vascular factors and AD focused on the mechanisms by which atherosclerosis of cerebral vessels and its effects on cerebral perfusion might contribute to AD pathology by increasing Aβ production and/or reducing its clearance from the brain (Iadecola, 2010). Recent evidence suggests that Aβ may also promote cerebrovascular atherosclerosis, resulting in a vicious circle whereby vascular damage and Aβ accumulation interact synergistically to increase brain dysfunction and damage. Our purpose here is to briefly review existing epidemiologic and clinical-pathological evidence which supports the coexistence and pathogenic overlap of AD and atherosclerosis. Since both large and small vessel pathology has been associated with AD, in addition to large-vessel atherosclerosis involving the large extracranial arteries and circle of Willis, we will also discuss microvascular pathology affecting smaller arteries and arterioles. Finally, we will examine pathways by which cerebrovascular damage might impair Aβ clearance and Aβ may promote atherogenesis, and their import for the treatment and prevention of AD.

## Evidence Supporting a Shared Link between Atherosclerosis and AD

### Epidemiologic Evidence and Imaging

Population-based epidemiologic data has significantly expanded our understanding that the pathogenesis of AD is broader in scope than might be apparent from neuronal studies focused exclusively on Amyloid Precursor Protein (APP) and Aβ. There is an extensive body of evidence showing that the risk factors for atherosclerotic vascular disease, including diabetes, hypercholesterolemia, and aging, are also risk factors for the development of AD (Breteler, 2000). For example, a prospective cohort study of over 1100 subjects without dementia at baseline showed that the presence of diabetes, hypertension, heart disease, and current smoking were associated with a higher risk of developing AD, and that the presence of three or more of these risk factors synergistically conferred a 3.4-fold increased risk of developing clinically apparent AD (Luchsinger et al., 2005). Additionally, in the Rotterdam Scan Study (Vermeer et al., 2003), in which 1015 participants were scanned with magnetic resonance imaging (MRI), investigators found that “silent” brain infarcts resulted in an almost 2.3-fold increased risk of dementia during a mean 3.6 year follow-up, with the majority of cases showing clinical evidence of AD.

More recent work has shown that specific vascular risk factors are not only predictive of clinical AD, but also of amyloid accumulation. Using an *in vivo* cerebral amyloid deposition measure with ^11^C-Pittsburgh compound B (PIB) positron emission tomography (PET), Reed et al. showed that an increased Framingham Coronary Risk Profile independently accounted for significant variance in amyloid deposition in the brain in a cross-sectional study in adults with normal cognition or mild cognitive impairment (Reed et al., 2012). In similar studies using PIB based imaging techniques, hypertension, high low-density lipoprotein cholesterol, and low high-density lipoprotein cholesterol have all been isolated as specific vascular risk factors that enhance amyloid deposition in the brain (Rodrigue et al., 2013; Reed et al., 2014). In light of such mounting data, in a recent analysis Barnes and Yaffe have suggested that up to half of the AD cases worldwide (over 17 million) are potentially attributable to vascular risk factors and that a 10–25% reduction of these risk factors could prevent over a million cases of AD cases worldwide (Barnes and Yaffe, 2011).

### Clinical-pathological Evidence

Additional clinical-pathological studies have shown a link between ischemic lesions and AD. For example, in the Nun Study (Snowdon et al., 1997), an autopsy study of the brains of 61 women with neuropathologic evidence of AD, those subjects with concurrent brain infarcts had poorer cognitive function and a higher prevalence of dementia than those without infarcts. In particular, participants who had lacunar infarcts in the basal ganglia, thalamus, or deep white matter had an almost 20-fold increased odds of dementia compared to those without infarcts. Conversely, in the 41 subjects without AD, the presence of brain infarcts was only weakly associated with cognitive impairment and dementia. Although the vascular pathology underlying the ischemic lesions was not established, these findings suggest that cerebrovascular disease plays a role in determining the clinical expression of AD. In a large cross-sectional study (Honig et al., 2005), the authors analyzed data from over 1000 subjects from the United States National Alzheimer’s Coordinating Center Database and found that atherosclerotic disease of the large cerebral arteries was strongly associated with neuritic plaque burden, a major pathologic manifestation of AD. However, other studies failed to report an association of AD pathology with cerebrovascular atherosclerosis (Dolan et al., 2010; Zheng et al., 2013), a discrepancy attributed to differences in patient selection (Chui et al., 2012). Nevertheless, the collective evidence lends support to the notion that ischemic brain lesions worsen the cognitive deficits in AD, and strongly suggest that vascular insufficiency and AD pathology are inextricably linked in the expression of the dementia (Iadecola and Gorelick, 2003; Toledo et al., 2013).

Further clinical studies have focused on more direct measures of atherosclerotic vascular disease. An autopsy study (Roher et al., 2003) of 32 AD patients and 22 nondemented controls analyzed atherosclerotic disease patterns in the circle of Willis. In this study, intracranial stenoses were determined based on histopathologic analysis of arteries cut into cross-sections and examined under a dissecting microscope for atheroma-related vessel narrowing. It was found that in AD patients the circle of Willis arteries exhibited significantly more numerous and severe intracranial stenoses than in nondemented controls. Moreover, this study found that a composite measure of the intracranial stenosis burden significantly correlated with multiple metrics of AD neuropathology, including the plaques, tangles, and Braak stage score (Roher et al., 2003). Larger subsequent neuropathological studies (Beach et al., 2007) confirmed the strong, independent association between intracranial atherosclerosis and AD after correcting for age, gender, and apolipoprotein E4 allele status. Most recently, in a large cohort of 1000 cases with gross and microscopic neuropathological data, investigators found that 77% of AD subjects had grossly apparent circle of Willis atherosclerosis, a rate which was significantly higher than that of normal subjects or of subjects with non-AD disease (Yarchoan et al., 2012). Although multiple histopathologic studies support a relationship between AD and large vessel atherosclerosis, the extent to which AD is associated with small vessel arteriolosclerotic changes, defined as concentrically thickened and/or dysmorphic arterioles, remains less clear. For example, a recent study failed to detect arteriolosclerosis in the frontal cortex of AD brains (Neltner et al., 2014). Another study demonstrated evidence of hypoxia-linked gene expression in the brain of patients with AD (Thomas et al., 2015). The effect was not associated with structural alterations of arterioles or amyloid angiopathy, but with increased expression of the vasoconstrictor endothelin-1. Although the contribution of hypoperfusion from large artery atheroma could not be assessed, these observations provide evidence that the brain of AD patients is under hypoxic stress, which has important implications for Aβ accumulation (see next section).

Beyond these clinical-pathological studies, modern structural neuroimaging allows for both direct and indirect assessment of macroangiopathic atherosclerotic disease (Gupta et al., 2012). Historically, *in vivo* clinical assessment of vascular abnormalities has relied on arterial catheterization and angiographic studies requiring the administration of iodinated contrast, which though highly accurate in assessing luminal narrowing, is invasive, carries significant potential risks, and cannot provide direct visualization of plaque or the arterial vessel wall. More recently developed techniques, now widely used in clinical practice, such as magnetic resonance angiography (MRA) and computed tomographic angiography (CTA), are cross-sectional imaging techniques for the visualization of major intracranial and extracranial arterial structures, and for delineation between vessel wall, vessel lumen, and atherosclerotic plaque (Gupta et al., 2012). In addition, techniques involving measurement of Cerebral Blood Flow (CBF) including CT and MR perfusion or intravascular flow velocities, such as transcranial Doppler and carotid sonography, allow for the detection of hemodynamic abnormalities that provide indirect evidence of vessel pathology, which in aging adults is most commonly related to atherosclerotic vessel narrowing (Gupta et al., 2012). In more recent work employing one such technique, Roher et al. (2011) used transcranial Doppler (TCD) ultrasound in 42 AD subjects and 50 nondemented controls to measure pulsatility indices of the large intracranial arteries of the circle of Willis, defined as the transcranial Doppler-determined peak systolic velocity minus the end-diastolic velocity, divided by the overall mean flow velocity (Roher et al., 2011). Confirming earlier studies (Ruitenberg et al., 2005), AD patients were found to have decreased arterial mean flow velocity and increased pulsatility index in the vascular territories supplied by the circle of Willis. This study also found that there were decreased diastolic flow velocities in the major extracranial arteries in the neck (the carotid arteries) in AD patients vs. nondemented controls, presumably related to loss of elastic capacity secondary to wall stiffening of large cerebral arteries.

In an attempt to link together physiologic measures of arteriosclerosis with imaging markers of AD pathology, investigators recently studied *in vivo* amyloid deposition and its relationship to arterial stiffness in 81 elderly nondemented individuals (Hughes et al., 2014). In this longitudinal cohort study, PET with PIB was used to evaluate for the presence of amyloid both at baseline and at a 2-year follow up scan. In addition, arterial stiffness was measured using a noninvasive automated waveform analyzer in both central and peripheral vascular beds. The investigation showed that the increase in proportion of amyloid positive subject from 48% to 75% was strongly associated with increases of arterial stiffness over time. Therefore, arterial stiffness is strongly associated with the progressive deposition of Aβ in the brain, a conclusion also supported by prior work showing associations between AD and peripheral vascular disease (Newman et al., 2005), and carotid artery intima-media thickness (Hofman et al., 1997; Gorgone et al., 2009). The interplay between CBF impairment, increased arterial rigidity, and AD has been confirmed by several similar investigations (Alsop et al., 2000; van Oijen et al., 2007; Dai et al., 2009; Jurasic et al., 2009; Silvestrini et al., 2009). These data, collectively, suggest that noninvasive assessments of macroangiopathic atherosclerosis with modern neuroimaging techniques might aid in identifying those patients potentially at greatest risk of developing dementia, including AD, and who could benefit most from strict vascular risk factor control and vasoprotective therapies.

## Mechanisms Linking Atherosclerosis and AD

### Vascular Insufficiency and APP Processing

Persistent brain hypoperfusion, as a result of both flow-limiting large vessel atherosclerotic stenoses and increased vascular stiffness resistance of the small and large vessels, has been linked to the pathophysiologic cascade leading to AD pathology. Brain hypoxia, which can result from advanced atherosclerosis, has been shown to significantly increase the cleavage of Aβ from the APP via upregulation of the β- and γ-secretase enzymatic pathways (Sun et al., 2006; Tesco et al., 2007; Zhang et al., 2007; Li et al., 2009). Experimental studies have confirmed the role of hypoperfusion in exacerbating amyloid pathology. For example, chronic cerebral hypoperfusion secondary to mechanically-induced bilateral common carotid artery stenosis in mice has been shown to cause an accelerated deposition of leptomeningeal Aβ and an increased incidence of cortical microinfarcts compared to mice without carotid stenosis (Okamoto et al., 2012). Consistent with a role of hypoxia-ischemic in promoting Aβ production, plasma Aβ has been reported to increase after cardiac arrest in humans (Zetterberg et al., 2011). Furthermore, experimental hypertension, which compromises cerebral perfusion, aggravates vascular dysfunction and enhances amyloidogenic APP processing by promoting β-secretase activity (Faraco et al., 2015).

The deleterious effects of Aβ on cerebrovascular function are well-characterized and include increased arterial vasoconstriction, reduction in resting CBF, attenuated endothelium-dependent vasodilatation, and reduction in the extent of CBF increase evoked by neural activity (Thomas et al., 1996; Niwa et al., 2000a,b, 2001; Iadecola, 2003a, 2004) Similar cerebrovascular dysfunction has been observed in mice in which brain Aβ is increased by overexpression of mutated APP (Iadecola et al., 1999; Niwa et al., 2002a,b). These observations have led to the hypothesis that Aβ in addition to its well established deleterious effects on neuronal activity, also impairs cerebral perfusion, setting the stage for ischemic brain injury (Iadecola, 2003b). In support of this hypothesis, mice overexpressing APP are more susceptible to cerebral ischemia (Zhang et al., 1997), whereas the incidence of ischemic stroke is increased in AD patients (Chi et al., 2013; Tolppanen et al., 2013). At the same time, as reviewed above, hypoxia may increase the rate of cleavage of Aβ from APP and tau phosphorylation (Koike et al., 2010). Therefore, the deleterious vascular effects of Aβ are likely to act synergistically with large artery atherosclerosis to aggravate cerebral perfusion, which, in turn, promotes amyloidogenesis by increasing APP cleavage.

### Atherosclerosis and Aβ Clearance

Though there has been significant focus on the increase in Aβ production caused by the downstream effects of atherosclerosis of the large and small cerebral arteries, there has been increasing evidence suggesting that impairment of Aβ clearance may also be an additional link between atherosclerosis and AD neuropathology. Mechanisms that play a central role in the clearance of Aβ from the brain include degradation via neprilysin and other Aβ-degrading enzymes, CSF reabsorption, and removal through vascular mechanisms, which include transfer into the bloodstream via the lipoprotein receptor-related protein-1 and perivascular drainage (Miners et al., 2008; Bell and Zlokovic, 2009). The perivascular pathway is involved in the drainage of soluble metabolites, proteins and peptides from the brain, which ultimately flows into the cervical lymph nodes (Hawkes et al., 2011; Carare et al., 2013). In this pathway, interstitial fluid enters the perivascular space surrounding the vascular basement membrane (Virchow-Robin Space) by bulk flow (Hawkes et al., 2014). Because such solute transport occurs in blood vessel walls in the opposite direction of blood flow, the pressure gradient driving such an exchange is uncertain. Furthermore, studying the molecular transports along these perivascular pathways has been challenging to investigate experimentally, given the small size and rapidity of such drainage *in vivo* (Hawkes et al., 2014). However, the relative contribution of the major Aβ clearance pathways has recently been estimated in humans (Roberts et al., 2014). It was found that 50% of Aβ clearance could be attributed to CSF and vascular-perivascular pathways (Roberts et al., 2014), attesting to the importance of vascular Aβ clearance also in the human brain.

Studies using mathematical modeling of perivascular solute transport in the brain strongly suggest that the driving force for such solute transfer is the recoil or reflection wave of the arterial wall after normal cardiac pulse wave (Schley et al., 2006; Wang and Olbricht, 2011). For example, in a model assuming that solute drainage occurs through a thin layer between astrocytes and endothelial cells (Schley et al., 2006), the successful drainage of solutes such as Aβ likely occurs in each pulse cycle during discrete, transient time periods when fluid and solutes are driven in the perivascular space in the opposite direction of blood flow. Moreover, in this model, it is likely that the efficient drainage of interstitial fluid requires some attachment of solutes to the lining of the perivascular space in order to produce a one-way valve effect. This model predicts that reductions in the amplitude of vessel wall movement in disease states, such as might be seen in hypoperfusion from proximal arterial stenosis or wall-stiffening in microangiopathic arteriosclerosis, will prolong solute attachment time to the perivascular space lining. In such a scenario, in the case of Aβ, for example, these reductions in the amplitude of vessel wall movement would reduce Aβ clearance and might therefore precipitate the type of vascular and parenchymal accumulation of Aβ seen in AD. In another analysis of fluid mechanics in the perivascular space (Wang and Olbricht, 2011), mathematical models also showed that the peristaltic motion of blood vessel walls are needed to facilitate fluid and solute transport in the perivascular space. These modeling data are consistent with the distribution of Aβ in the walls of large cortical and leptomeningeal arteries in cerebral amyloid angiopathy which support the notion that Aβ is likely deposited in the perivascular pathways of interstitial fluid drainage from the brain (Hawkes et al., 2014). Experimental models with mice also support this hypothesis, with experimental data (Hawkes et al., 2011) suggesting that the Aβ injected into the interstitial fluid of adult mice distribute in the basement membrane in the same pattern as cerebral amyloid angiopathy in humans, which can in turn further impair perivascular drainage in the brain.

More recently, new MRI technology has been used to demonstrate *in vivo* the perivascular pathways, which exist for CSF and interstitial fluid exchange that enable solute clearance from the brain. For example, using dynamic contrast-enhanced MRI of the rat brain, a recent study confirmed that CSF flow occurs through major para-arterial pathways and that exchange between the CSF and interstitial space occurs in molecular-size dependent fashion (Iliff et al., 2013). Similarly, using both *in vivo* and *ex vivo* fluorescence microscopy, in a separate study investigators studied interstitial solute clearance after Aβ injection into the brain parenchyma of mice (Kress et al., 2014). Compared to relatively young wild-type mice, the clearance of Aβ from the brain parenchyma was impaired by 40% in old wild-type mice. Importantly, these investigators found a 27% reduction in vessel wall pulsatility of intracortical arterioles associated with the decline in Aβ solute exchange, suggesting that arterial wall stiffening may play a central role in allowing for the successful clearance of Aβ. Whether impaired clearance of Aβ may be occurring secondary to myocyte loss in the absence of thrombotic plaques *per se* requires further investigation, potentially with similar advanced MRI techniques using animal models.

When these animal and theoretical modeling data are viewed in light of the human clinical and epidemiologic evidence linking AD and atherosclerosis, impaired clearance of Aβ via the perivascular drainage pathways becomes a highly plausible, additional mechanism by which Aβ may exert deleterious effects on brain vascular biology. Both distal flow reduction from macroangiopathic atherosclerotic narrowing (such as would be seen with carotid stenosis) and vascular stiffening would be plausible mechanisms by which atherosclerosis could impair the driving force needed to efficiently clear Aβ via the perivascular routes. Clearly, until methods of measuring Aβ clearance in humans (Roberts et al., 2014) are applied more widely, much of the evidence supporting this relationship between atherosclerosis and Aβ clearance remains largely inferential and requires additional study. Moreover, potential mechanisms for improving Aβ clearance, such as the use of vasoactive agents that increase arterial pulsatility (Iliff et al., 2013), represent novel therapeutic strategies also worthy of further investigations.

While more precise diagnostic and therapeutic strategies focused on Aβ clearance are being studied, on a broader scale, improving our understanding of the mechanisms linking atherosclerosis with AD remains critically important. Such work should serve to increase the urgency and resources with which cardiovascular diseases in general are treated, given that atherosclerosis remains both underdiagnosed and undertreated (Bhatt et al., 2006). Preliminary work to date has been promising, with studies suggesting that control of vascular risk factors may reduce MRI defined white matter lesions in AD and, at least early in the disease course, may delay AD progression (Rosenberg et al., 2008; Deschaintre et al., 2009; Richard et al., 2010; Valenti et al., 2014). Furthermore, the recently reported reduction in the incidence of AD has been attributed in large part to a better control of vascular risk factors (Larson et al., 2013).

### Aβ and Atherosclerosis

Whereas, as discussed above, atherosclerosis and related vascular insufficiency can promote Aβ accumulation, there is converging evidence suggesting that Aβ can promote atherogenesis. Autopsy data from human brains have shown that amongst non-demented elderly subjects, a high burden of atherosclerosis in the circle of Willis is strongly associated with increased levels of Aβ42 compared to subjects with lesser degrees of cerebral atherosclerosis, even though levels of β-secretase are comparable between high and low-atherosclerosis groups (Sadleir et al., 2013). Though the cause of such brain Aβ elevations is uncertain, several lines of investigation suggest that Aβ itself is atherogenic. Early work supporting this hypothesis includes the finding that Aβ and APP are found in the microvasculature surrounding advanced human carotid artery plaque (De Meyer et al., 2002). The latter study also found that platelet-derived APP is proteolytically converted to Aβ and that this pathway results in inducible nitric oxide synthase induction and increased macrophage activation, findings which suggest that Aβ may play a key role in the pro-inflammatory cascade of atherosclerosis. More recent work in mouse models (Tibolla et al., 2010; Caravaggio et al., 2013) has further supported the role of Aβ in the development of atherosclerotic plaque. Caravaggio et al. (2013) for example, used an insulin-degrading enzyme and lipoprotein receptor-deficient mouse model to show that insulin-degrading enzyme deficiency results not only in larger atherosclerotic lesions but also increased levels of Aβ within these plaques. Similarly, the work of Tibolla et al. (2010) in which a mouse model with central nervous system-restricted APP overexpression was studied, suggests that the inflammatory effects of APP overexpression can lead to aortic atherosclerosis even before brain parenchymal Aβ deposition. These findings are consistent with the hypothesis that while atherosclerosis-induced vascular insufficiency increases Aβ production and accumulation, Aβ, in turn, promotes atherogenesis (Iadecola, 2003a). However, the available data is still limited and future studies are warranted to further assess the mechanisms by which Aβ promotes atherosclerotic plaque formation in order to improve our understanding of the causal role which Aβ plays in atherogenesis.

## Conclusions

**Figure 1:**
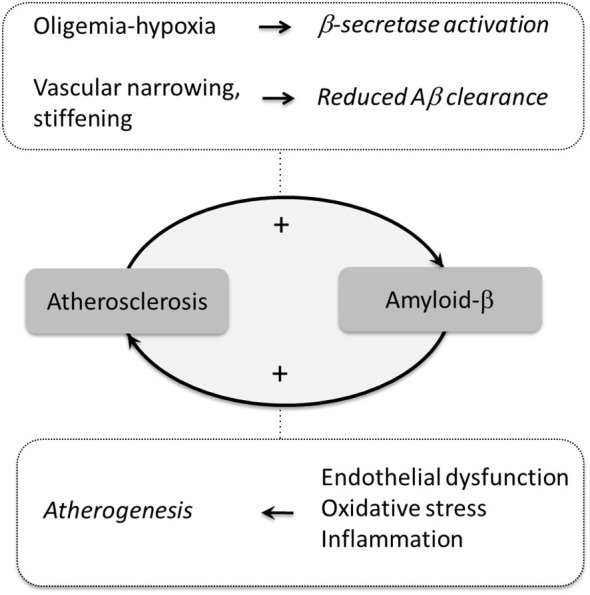
**Potential mechanisms linking atherosclerosis to amyloid pathology**. Atherosclerosis promotes brain Aβ accumulation by reducing cerebral blood flow (oligemia) leading to hypoxia, which increases Aβ production by activating β-secretase. In turn, Aβ promotes atherogenesis through endothelial dysfunction, oxidative stress, and inflammation.

Atherosclerosis and AD, independently, are two of the largest public health burdens in aging populations, and together, appear to act in an additive or potentially synergistic fashion based on mounting epidemiologic, clinical and experimental evidence (Figure 1). The vascular biology of Aβ figures prominently in most theories linking atherosclerosis with the development and progression of AD. While increased production of Aβ may link cerebral hypoperfusion to AD, impaired clearance of Aβ has emerged an additional mechanism, which may be occurring simultaneously and act in concert to enhance Aβ deposition. Thus, large vessel flow-limiting stenosis, macro- and micro-angiopathic arterial stiffening and neurovascular dysregulation could also play a role in AD pathophysiology by aggravating the hypoperfusion, impairing Aβ clearance and elevating brain levels of Aβ. A link between Aβ and the process of atherogenesis is also emerging, whereby Aβ could promote formation of atherosclerotic lesions leading to additional vascular damage. Further studies are now warranted to investigate this recently appreciated mechanistic link between AD and cerebrovascular disease, and new methods to investigate Aβ deposition and trafficking in the human brain promise to advance the field forward. In the meantime, in the absence of effective therapies to target Aβ, tau and their deleterious effects, the evidence suggests that the optimal management of vascular risk factors may be the most beneficial means of reducing the morbidity not only of cardiovascular ischemic events, but AD as well. This course of action has recently been validated by a study in which diet, exercise, and vascular risk factor monitoring were found to slow down the progression of dementia (Ngandu et al., 2015).

## Conflict of Interest Statement

The authors declare that the research was conducted in the absence of any commercial or financial relationships that could be construed as a potential conflict of interest.
